# *Omega-1* knockdown in *Schistosoma mansoni* eggs by lentivirus transduction reduces granuloma size *in vivo*

**DOI:** 10.1038/ncomms6375

**Published:** 2014-11-17

**Authors:** Jana Hagen, Neil D. Young, Alison L. Every, Charles N. Pagel, Corinna Schnoeller, Jean-Pierre Y. Scheerlinck, Robin B. Gasser, Bernd H. Kalinna

**Affiliations:** 1 Faculty of Veterinary and Agricultural Sciences, The University of Melbourne, Parkville, Melbourne, Victoria 3010, Australia; 2 Department of Life Sciences, Imperial College London, London SW7 2AZ, UK

## Abstract

Schistosomiasis, one of the most important neglected tropical diseases worldwide, is caused by flatworms (blood flukes or schistosomes) that live in the bloodstream of humans. The hepatointestinal form of this debilitating disease results from a chronic infection with *Schistosoma mansoni* or *Schistosoma japonicum*. No vaccine is available to prevent schistosomiasis, and treatment relies predominantly on the use of a single drug, praziquantel. In spite of considerable research effort over the years, very little is known about the complex *in vivo* events that lead to granuloma formation and other pathological changes during infection. Here we use, for the first time, a lentivirus-based transduction system to deliver microRNA-adapted short hairpin RNAs (shRNAmirs) into the parasite to silence and explore selected protein-encoding genes of *S. mansoni* implicated in the disease process. This gene-silencing system has potential to be used for functional genomic–phenomic studies of a range of socioeconomically important pathogens.

## Abstract (web summary)

Schistosomiasis, a neglected tropical disease, is caused by flatworms such as *Schistosoma mansoni*. Here, Hagen *et al*. describe a lentivirus-based transduction system to deliver microRNA-adapted small hairpin RNAs into *S. mansoni* to inhibit transcription of selected genes implicated in the disease process.

## Graphical abstract

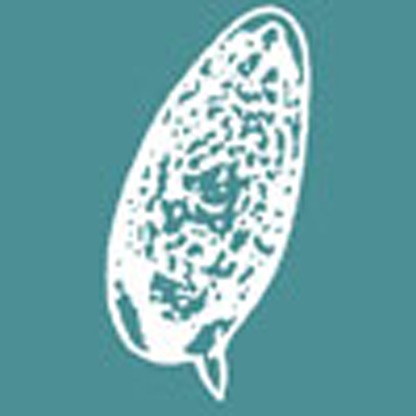

Schistosomiasis is among the most important neglected tropical diseases worldwide, affecting ~200 million people globally and causing 300,000 deaths per annum^1^. The hepatointestinal form of this debilitating disease is usually caused by a chronic infection with *S. mansoni* or *S. japonicum* (blood flukes). No vaccine is available, and treatment relies on the use of one drug (praziquantel), to which resistance is emerging. Of the three main species of schistosome that infect people, *S. mansoni* is widespread throughout Africa, South America and the Caribbean^1^. Through a complex aquatic life cycle, *S. mansoni* is transmitted (via skin penetration) from an infected, aquatic snail (*Biomphalaria* spp.) to humans. Adult worms dwell in hepatic and intestinal vessels, where they release eggs that become embedded in the liver or intestinal wall, and trigger immune-mediated granuloma formation and associated clinical complications, such as periportal fibrosis and hypertension^2^. Although some immunopathological changes linked to the granulomata have been studied^2^, there is limited knowledge of the precise mechanisms underlying these alterations. This knowledge gap relates mainly to the complexity of the parasite life cycle and technical obstacles.

Underpinned by major advances in our understanding of schistosome genomes^3^,^4^, gradual progress in the development of functional genomic tools^5^,^6^,^7^,^8^ provides new opportunities to gain insights into the intricacies of the schistosome–host relationship. Various tools, such as RNA interference (RNAi) using double-stranded RNA or short interfering RNA (siRNA), have been used to investigate the functions of single genes; however, some techniques employed to date can have limitations, such as off-target (that is, nonspecific) effects and an inadequate persistence of gene knockdown for subsequent phenotypic assessment *in vitro* or *in vivo* in the host(s)^9^. These issues are compounded by the challenges of consistently producing sufficient amounts of appropriate parasite stages for functional genomic analyses^10^. Nonetheless, recent evidence has shown great promise for the use of a lentivirus-based transduction system for specific and persistent gene knockdown in mammalian cells^11^, with the potential of overcoming most of the disadvantages of previous knockdown methods.

Here we show how lentivirus transduction can be used to achieve specific and persistent knockdown of selected genes (*omega-1*, *ipse* and/or *kappa-5*)^12^ of *S. mansoni*, implicated in egg-induced granulomatous responses in the mammalian host^13^,^14^. This study allows, for the first time, the functional genomic–phenomic exploration of schistosomiasis mansoni *in vivo.*

## Results

### Lentivirus transduction

**Figure 1:**
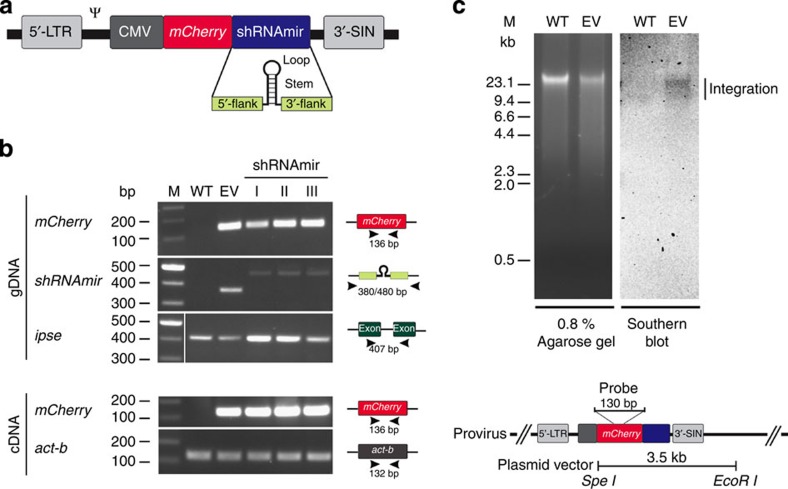
Verification of transgene and transcription in eggs of *S. mansoni* following transduction with lentivirus constructs. (**a**) Lentivirus construct encoding shRNAmir/*mCherry* expression cassette under the control of a CMV promoter and containing a packaging signal (Ψ) as well as a long terminal repeat (5′-LTR) and a self-inactivating (3′-SIN) LTR sequence. The shRNAmir structure is indicated. (**b**) Genomic DNA (gDNA) and RNA were isolated from eggs 10 days after transduction. Lentiviral DNA was detected in gDNA by direct PCR amplification of transgene regions, using the gene *ipse* as a positive control; cDNA was produced by reverse transcription from total RNA from eggs. Transcription of the transgene was demonstrated by direct PCR of *mCherry* transgene regions from cDNA, employing the *actin-β* (*act-b*) coding region as a positive control (cf. Supplementary Fig. 1 for full gel images). (**c**) Representative Southern blot analysis of gDNA from eggs of *S. mansoni* (3 days after transduction) confirming genomic integration of the lentivirus by specific hybridization (vertical bar). For this analysis, gDNA was isolated from eggs 3 days after lentivirus transduction, digested with both restriction endonucleases *Spe*I and *Eco*RI (the plasmid vector contains only one site for each enzyme); 5 μg of digested DNA were resolved in an agarose gel (0.8%) and subjected to specific hybridization using a probe (see inset) directed to the transgene encoded by the lentivirus. The expected sizes of the empty vector (EV; plasmid) control and any non-integrated viral DNA were 3.5 and 5.5/2.1 kb, respectively. Lanes: M, molecular weight marker in bp; WT, wildtype; EV, EV control; I, shRNAmir-511+shRNAmir-557 to *omega-1*; II, shRNAmir-195+shRNAmir-384 to *ipse*; III, shRNAmir-616+shRNAmir-638 to *kappa-5*; shRNAmir, microRNA-adapted short hairpin RNA.

First, we transduced *S. mansoni* eggs with lentiviral constructs encoding microRNA-adapted short hairpin RNAs (shRNAmirs) (Fig. 1a). Ten days after transduction with lentiviral particles, both regions of the *mCherry* gene and regions encoding individual shRNAmirs were detected in the genomic DNAs of treated eggs, whereas neither of these regions was found in DNA from unexposed eggs (Fig. 1b), indicating successful delivery of viral DNA into the nucleus of the host cell. Southern blot hybridization confirmed proviral integration into the genome of *S. mansoni* (Fig. 1c). Moreover, *mCherry* was transcriptionally active under the control of the cytomegalovirus (CMV) promoter, as transcripts were detected in complementary DNA from virus exposed but not from unexposed eggs (Fig. 1b). Although attempts to detect fluorescence and assess transduction efficiency in transduced eggs were impaired by autofluorescence of the eggshell (not shown), results demonstrated successful lentiviral transduction and CMV-driven transgene expression in *S. mansoni* eggs.

### shRNAmirs silence transcription in *S. mansoni in vitro*

**Figure 2:**
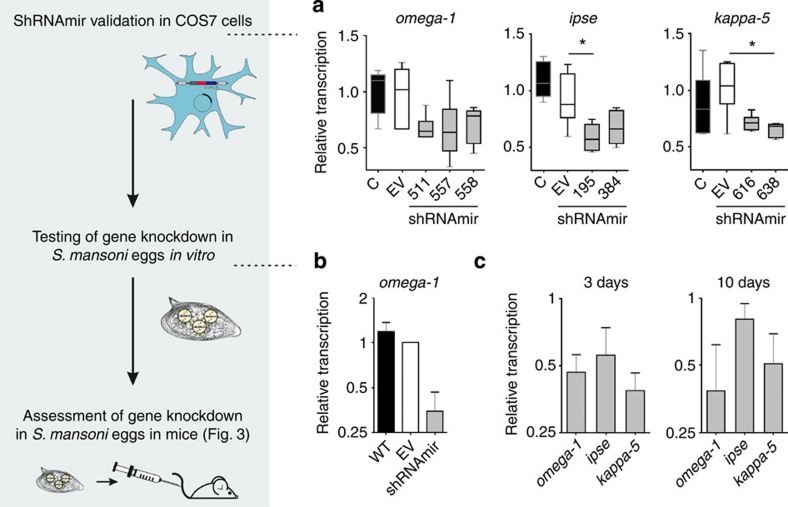
Validation of artificial microRNAs in COS7 cells and lentivirus-based transduction of *S. mansoni* eggs. (**a**) Validation of shRNAmir lentivirus constructs in COS7 cells transfected with plasmids encoding *omega-1*, *ipse* or *kappa-5*. COS7 cells were either not transfected (C) or transfected with vector plasmids without the shRNAmir stem-loop sequence (that is, empty-vector (EV)) or with vector plasmids containing gene-specific shRNAmirs shRNAmir-511, shRNAmir-557 and shRNAmir-558 (*omega-1*); shRNAmir-195, shRNAmir-384 (*ipse*), shRNAmir-616 and shRNAmir-638 (*kappa-5*) (labelled with numbers only). Transcription was assessed by qPCR after 48 h, using *act-b* as a reference gene. Kruskal–Wallis and Dunns *post*-*hoc* tests were used to establish statistical significance compared with the EV control: *P<0.05; *n*=6. (**b**) Downregulation of *omega-1* transcription in *S. mansoni* eggs 3 days after transduction with a single shRNAmir-containing lentivirus (shRNAmir-557) were compared with untreated eggs (wild type (WT) control) and eggs transduced with EV lentivirus. Relative transcription of *omega-1* in eggs was assessed by qPCR 3 days after transduction; transcription was normalized against the geometric mean of Ct values for three reference genes (*psdm4*, *ef1-a* and *cox-1*). Bars show mean±s.e.m.-normalized transcription for untreated control eggs or *omega-1*-knockdown samples relative to the EV control eggs for three independent experiments. (**c**) Transcription of *omega-1*, *ipse* or *kappa-5* was assessed by qPCR 3 or 10 days following transduction. Bars show the mean±s.e.m.-normalized transcription for biological replicates (*n*=3) of shRNAmir-treated eggs of the same experiment, relative to the EV control.

Before testing in *S. mansoni* eggs, we assessed the functionality of seven shRNAmirs in mammalian (COS7) cells expressing cDNA of the target. Treatment with shRNAmir-511, shRNAmir-557 or shRNAmir-558, targeting the *omega-1* gene, showed a 29–34% decrease in transcription compared with ‘empty-vector’ (EV) control samples; the transcription of the *ipse* and *kappa-5* genes was consistently reduced by 33–42% (shRNAmir-195 or shRNAmir-384) and 28–34% (shRNAmir-616 or shRNAmir-638), respectively, following treatment (Fig. 2a). The subsequent transduction of *S. mansoni* eggs with lentivirus encoding shRNAmir-557 and targeting transcripts of *omega-1* led to a 45–85% decrease in transcription compared with EV control samples in three independent experiments (Fig. 2b); transcription in EV-treated eggs did not differ from that of untreated control samples. This result shows, for the first time, that shRNAmirs are functional in *S. mansoni* and can be used for RNAi. Next, we assessed gene knockdown in *S. mansoni* eggs using a combination of two viruses encoding different shRNAmir sequences that targeted *omega-1* (shRNAmir-511+shRNAmir-557), *ipse* (shRNAmir-195+shRNAmir-384) or *kappa-5* (shRNAmir-616+shRNAmir-638). Three days after viral transduction, a 50–60% reduction in transcription was recorded for all shRNAmir treatment groups compared with EV controls (Fig. 2c); other results showed that the knockdown effect on target gene transcription persisted for at least 10 days following transduction (Fig. 2c). Importantly, lentiviral transduction and shRNAmir-induced knockdown of transcription had no effect on the maturation or vitality of miracidia in eggs (Supplementary Movie), providing the basis for an evaluation of transduced eggs *in vivo* in mice.

### Effect of knockdown on egg–induced pathology

**Figure 3:**
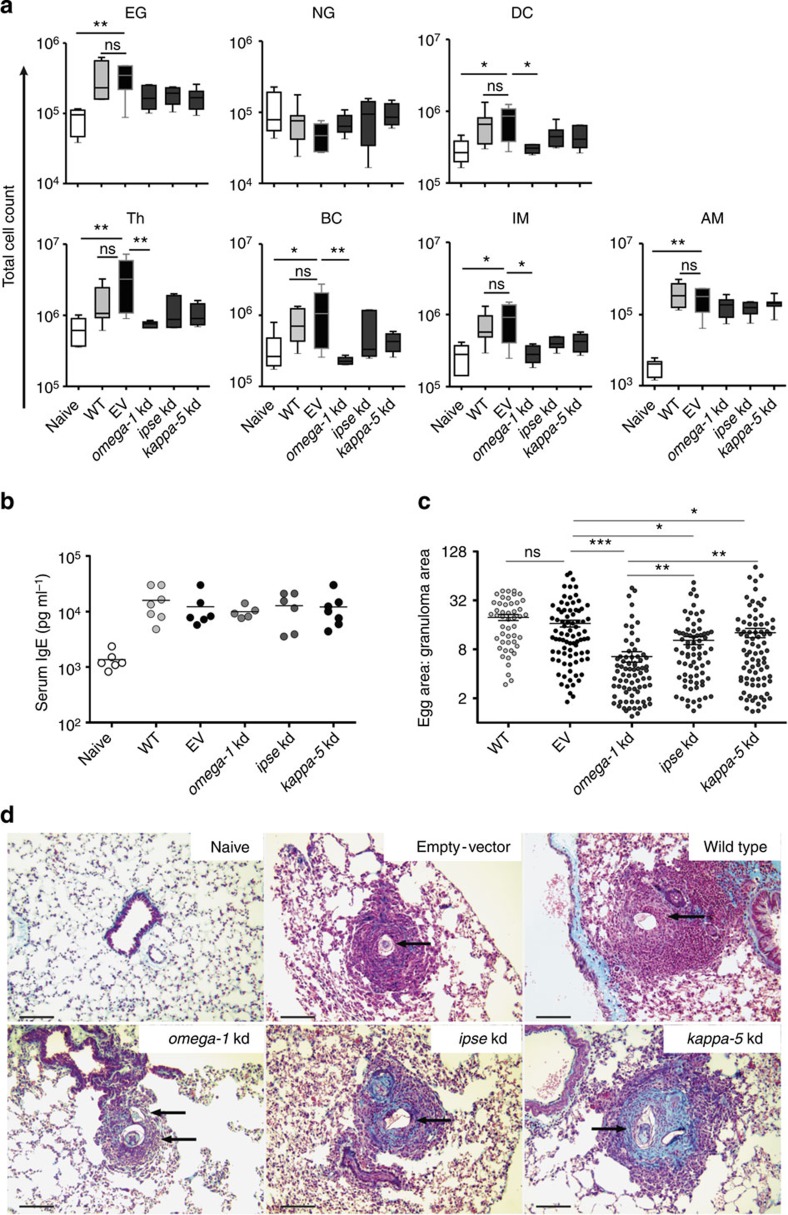
Pathological changes in the lungs of BALB/c mice injected with *omega-1*, *ipse* or *kappa-5* knockdown (kd) eggs of *S. mansoni*. Test mice were injected into the lateral tail vein with 1,000 eggs previously transduced with lentiviruses containing gene-specific shRNAmir stems (*omega-1*, *n*=5; *ipse, n*=6; *kappa-5; n=*7). Control groups of mice received PBS (naive; *n*=6), 1,000 untreated wild type eggs (WT; *n*=7) or 1,000 eggs previously transduced with a lentivirus lacking the shRNAmir stem (that is, empty-vector (EV); *n*=6). All mice were euthanized 15 days after injection. (**a**) Total leukocyte numbers in cell suspensions from the right lung; cell populations: eosinophil granulocytes (EG), neutrophil granulocytes (NG), dendritic cells (DC), T-helper cells (Th), B cells (BC), interstitial macrophages (IM) or alveolar macrophages (AM). The percentage of leukocyte populations was established by flow cytometry (see Supplementary Fig. 2 for cell gating) and calculated as the total cell number of live cells isolated from the right lung from individual mice. Box plot representing the median and upper/lower quartile. Whiskers indicate the highest or lowest value. Kruskal–Wallis and Dunns *post-hoc* tests were used to establish statistical significance compared with the EV control: **P<0.01. (**b**) Effect of *omega-1*, *ipse* or *kappa-5* knockdown of *S. mansoni* eggs on total serum IgE levels in mice 15 days following injection of eggs. (**c**) Effect of egg knockdown (kd) on granuloma formation in the left lung. Granuloma sizes were determined as the ratio of the granuloma area to the egg area. Scatter plot with mean±s.e.m. of the data pool representing all granulomata from all lungs from each experimental group (WT: *n*=48; EV: *n*=82; *omega-1* kd: *n*=77; *ipse* kd: *n*=74; *kappa-5* kd: *n*=84). Kruskal–Wallis and Dunns *post-hoc* tests were used to establish statistical significance compared with the EV control: *P<0.05, **P<0.01, ***P<0.001; not significant (ns). Numbers of mice: *n*=4 (WT); *n*=5 (EV, *omega-1* kd); *n*=6 (*ipse* kd) and *n*=7 (*kappa*-5 kd). (**d**) Mean granuloma size in left lung sections from mice examined microscopically (original magnification: × 200), following staining with Masson–Goldner Trichrome. Scale bars, 100 μM. Arrows indicate *S. mansoni* eggs.

**Figure 4:**
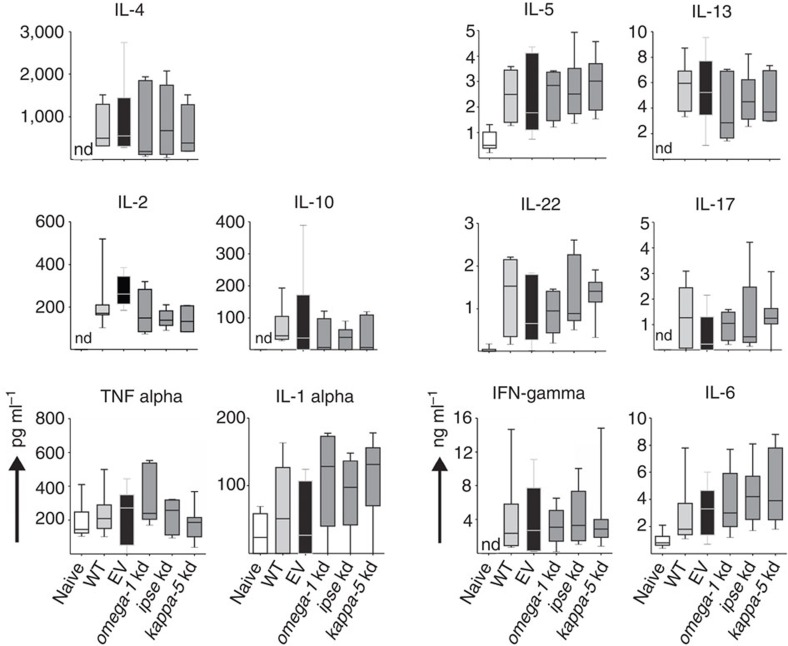
*Ex vivo* cytokine production of lung cell suspensions after antigen-specific restimulation. Lung cells of BALB/c mice were analysed for cytokine secretion 15 days after injection, via the tail vein, with PBS (naive), eggs of *S. mansoni* treated with no virus (WT), empty-vector (EV), or lentiviruses containing shRNAmirs directed at *omega-1*, *ipse* or *kappa-5* transcripts. Lung cell suspensions were restimulated with SEA (20 μg ml^−1^) for 72 h and culture supernatants (*n*=5–7) assayed by cytometric bead array. Box plot representing the median and upper/lower quartile. Whiskers indicate the highest or lowest value. IL, interleukin; IFN, interferon; kd, knockdown; nd, not detected.

As omega-1, IPSE (egg-secreted cytotoxic or immunomodulatory glycoproteins) and kappa-5 (non-secreted protein) have been implicated in egg-induced responses^12^,^14^, we investigated whether knockdown of genes encoding each of these egg proteins had an effect on the development of granulomata associated with pulmonary schistosomiasis induced in BALB/c mice following the injection of *S. mansoni* eggs into the tail vein. In this established experimental mouse model, eggs are transported to the lungs via the bloodstream and pass into the lung tissues where they become embedded and induce immune responses, leading to subsequent granuloma formation that peaks ~2 weeks after injection^15^. In our investigation, there was a reduction in the numbers of dendritic cells (DCs), T-helper and B cells, as well as interstitial macrophages in the lungs of mice injected with *omega-1* knockdown eggs compared with those injected with eggs carrying the EV (Fig. 3a); the numbers were consistent with those of naive mice. The number of alveolar macrophages in the *omega-1* knockdown group was similar to that of the EV control mice, but elevated compared with naive mice (Fig. 3a). A slight decrease in infiltrating interstitial macrophages was also observed in *ipse* and *kappa-5* knockdown mice, but was not significantly different from the EV control mice (Fig. 3a). Interestingly, the numbers of eosinophil granulocytes infiltrating the lung were not affected by knockdown of any of the target genes of *S. mansoni* (Fig. 3a). Moreover, lung-associated neutrophil numbers were not altered in any of the treatment groups compared with naive control mice (Fig. 3a). The numbers of leukocyte infiltrates in the lung tissue did not differ significantly between wild type (WT) and EV-transduced, egg-injected control mice. Serum samples from mice that had been injected with WT or control virus-transduced eggs had an expected, significant increase in IgE levels, typical of *S. mansoni* infection; an increase was also seen in all egg-protein-knockdown groups of mice (Fig. 3b). Following a soluble egg antigen (SEA)-specific re-stimulation of lung cell isolates (Fig. 4), all egg-injected mice responded with a characteristic, dominant Th2 cytokine pattern compared with the naive control group. However, no significant difference was observed in any of the knockdown groups compared with EV or WT egg-injected mice.

Finally, we assessed whether gene knockdown for any of the three egg proteins had an effect on granuloma formation in the lungs of mice (Fig. 3c). Knockdown of *omega-1* in *S. mansoni* eggs led to a highly significant (*P*<0.001) decrease in granuloma size compared with the EV and untreated, WT control groups, with mean±s.e. of granuloma/egg ratios of 6.505±0.963, 19.75±1.685 and 16.63±1.597, respectively. Furthermore, granulomata in the *omega-1* knockdown group (6.505±0.963) were significantly (*P*<0.01; Kruskal–Wallis and Dunns *post-hoc* tests) smaller than those in *ipse* (10.31±1.162) and *kappa-5* (12.77±1.648) knockdown groups. No significant differences in granuloma size were measured between mice injected with WT (19.75±1.685) or control virus-treated eggs (16.63±1.597; Fig. 3c). Granulomata found in the lungs of the *kappa-5* knockdown group exhibited an increase in collagen deposition (Fig. 3d).

## Discussion

Here we demonstrated lentiviral transduction of *S. mansoni* eggs for the delivery of a shRNAmir expression cassette. This approach probably overcomes some of the limitations of conventional RNAi (for example, by soaking or electroporation), including a lack of persistent knockdown over a longer period of time (which is required for *in vivo* application in animals) and possible off-target effects of RNAi triggers. Although double-stranded RNA and siRNA usually achieve knockdown for periods of up to 4 weeks in *S. mansoni in vitro*^16^,^17^, it has been shown that siRNA does not induce a long-term effect *in vivo* when schistosomules are re-introduced into the definitive host^17^. The findings of the present study show that the integration of a lentivirus encoding an RNAi-trigger expression cassette circumvents this limitation to achieve sustained knockdown in *S. mansoni in vivo* in mice. The results obtained using this method also indicate higher specificity of gene knockdown compared with conventional RNAi. In contrast to previous γ-retrovirus-based methods employed to deliver shRNAs to *S. mansoni*^18^,^19^,^20^, the lentiviral delivery system can transduce both arrested and dividing cells to maximize knockdown efficiency. Furthermore, the expression of shRNAs as artificial primary miRNAs with a Drosha and Dicer processing sites has been shown to diminish the cytotoxic effect of a given short hairpin RNA (shRNA)^21^. The thermodynamic design of hairpins allows for a biased incorporation of the antisense strand into the RNA-induced silencing complex^22^. This approach decreases the risk of off-target effects induced by the sense (miRNA*) strand and enables off-target gene predictions to be made based on the antisense (miRNA) strand sequence. Moreover, the expression of shRNAs as shRNAmirs can increase target gene knockdown by up to tenfold^23^. Therefore, the use of more efficient shRNAmirs might also allow the application of weak promoters to minimize possible cytotoxicity linked to a saturation of the RNAi pathway^24^,^25^, and of low virus copy numbers to reduce the risk of adverse effects due to viral integration and promoter/enhancer interactions with endogenous genes, as shown previously for γ-retroviruses^26^. To avoid adverse effects resulting from an abundance of the RNAi trigger, shRNAmir expression levels need to be controlled. Such controlled expression of the RNAi trigger can be realized by using pol II-driven shRNAmir expression cassettes (as shown here), as pol II promoter activity can be constrained by regulatory elements^27^ or tissue-specific endogenous miRNAs^28^.

Using the present lentiviral transduction system, we demonstrated that *omega-1* knockdown in *S. mansoni* eggs led to a significant decrease in granuloma size in mice. The increased infiltration of DCs, T-helper and B cells, and interstitial macrophages seen in the lungs of EV control mice was consistent with that expected in egg-induced disease^29^ and was clearly abrogated by *omega-1* knockdown. Interestingly, although a decrease in B and T cells was detected in *omega-1* knockdown groups, the cytokine profile remained unaltered following re-stimulation with SEA. This finding might relate to the fact that cell numbers in the re-stimulation assay were normalized against leukocyte numbers and not against total cell numbers in lung cell suspensions. Nonetheless, considering the largely diminished infiltration of effector cells in mice injected with *omega-1* knockdown eggs, it is likely to be that the inflammatory milieu in the lungs is reduced compared with WT and EV control groups. However, the diminished infiltration of B and T cells, as well as macrophages, raises questions as to which cells are responsible for the cytokine secretion and how these cells are activated. Candidate innate immune cells are eosinophils (interleukin (IL)-4 and IL-13), mast cells (IL-1, IL-6 and IL-13) and basophils (IL-4 and IL-13). Moreover, natural killer T cells have been reported as a source of IL-4 and IL-13 (ref. 30). The data from the present and other studies of granuloma formation^31^,^32^,^33^ indicate that, although an anti-inflammatory environment can be established in the absence of CD4+T cells, Th2 effector cells are required to boost the IL-4 and IL-13 dominant cytokine milieu to induce granuloma formation.

The present study also suggests that the cytotoxicity of omega-1 (ref. 34) is a critical factor in the initiation of granuloma formation, leading to tissue destruction and activation of an innate immune response, which is maintained and amplified by Th2 cells. Indeed, Loke *et al*.^33^ showed that alternative activation is an innate response to injury that requires CD4+ T cells to be sustained during a chronic infection. Therefore, the change in macrophage numbers on knockdown suggests a new role for omega-1 relating to the recruitment of macrophages into tissues and involvement in fibrosis/granuloma formation^35^. Alternatively activated macrophages (AAMs) play a critical role in the prevention of severe disease development during *S. mansoni* infection, as they are essential for both wound-healing processes^36^ and the suppression of severe fibrosis^37^. Furthermore, macrophage-derived transforming growth factor-β1 has been linked to the activation of collagen production associated with healing and fibrosis^38^,^39^. Moreover, AAMs upregulate expression of the mannose receptor^40^, the receptor responsible for the uptake of omega-1 by DCs and its modulatory effect on DCs^41^. Thus, it would be informative to investigate whether omega-1 can modulate AAMs to promote their immunosuppressive effect(s) after binding to the mannose receptor.

Despite the diminished cell infiltration into lung tissues, granuloma formation was not entirely abolished in the *omega-1* knockdown group. A possible explanation is an incomplete suppression of omega-1 expression in eggs linked to the formation of small granulomata. Interestingly, IPSE has also been shown to be cytotoxic^42^ and might be responsible for the induction of smaller granulomata in the *omega-1* knockdown group. However, as IPSE is the most abundant egg-secreted protein^43^,^44^, it is conceivable that, in the case of an assumed IPSE-induced cytotoxicity, the impact on granuloma formation might have been more severe. A possible scenario is that, in the absence of omega-1, IPSE can activate basophils^45^ to secrete IL-4 and IL-13 at levels that are not excessive, because they are not amplified by Th2 effector cells. Therefore, IPSE might contribute to wound-healing processes to counter-regulate minor tissue damage caused by translocating eggs. The moderate knockdown of *ipse* transcripts and the observed phenotype might reflect the presence of multiple copies of the *ipse* gene in the genome of *S. mansoni*.

Interestingly, we found that large granulomata induced by *kappa-5* knockdown eggs contained more collagen compared with the WT and EV control groups (*cf*. Fig. 3d). Although the collagen content in tissues was not quantified, the data suggest that kappa-5 has a modulatory, anti-fibrotic effect and is involved in the regulation of fibrosis. Owing to its molecular weight, kappa-5 is retained within schistosome eggs^46^ and might only become accessible to the immune system when eggs disintegrate. However, in the present study, both the eggshell and the miracidium appeared to be intact, contradicting this theory. As kappa-5 can bind to C-type lectin receptors^47^, it would be interesting to assess whether this protein has a modulatory effect on macrophages that regulates collagen synthesis. Although kappa-5 is the only molecule presently known to induce IgE responses in people with schistosomiasis^46^, *kappa-5* knockdown did not lead to a reduction in total serum-IgE levels in mice in the present study (Fig. 3b). However, an effect on kappa-5-specific serum IgE levels cannot be excluded, such that further work is warranted to address this aspect.

In conclusion, using a functional genomic–phenomic approach, the present study provides evidence that a key mechanism underlying *S. mansoni* egg-induced pathological changes is tissue destruction caused by omega-1, facilitated by its immunomodulatory capacity. This study paves the way for future investigations of schistosomiasis using complementary post-genomic tools. Clearly, the lentivirus transduction system applied here, for the first time to any parasite, has major potential to be used broadly for functional genomic–phenomic investigations of socioeconomically important eukaryotic parasites.

## Methods

### Maintenance of *S. mansoni*

All experiments were approved by the Animal Ethics Committee of the University of Melbourne and performed in quarantine-approved facilities. The NMRI strain of *S. mansoni* was maintained in *Biomphalaria glabrata* (NMRI strain, NIH-NIAID Schistosomiasis Resource Center, USA; http://www.schisto-resource.org) and female, specific pathogen-free BALB/c mice (7–8 weeks of age)^48^.

### Lentivirus vector and production

The lentivirus vector pGIPZ_*mCherry* was based on pGIPZ (Open Biosystems) in which shRNA constructs are expressed as human microRNA-30 (miR30) primary transcripts (shRNAmir). The CMV turboGFP was substituted (via the *Xba*I and *Not*I sites) with the *mCherry* reporter gene sequence from pFPV*mCherry*^49^. The CMV promoter was inserted into the pGIPZ*mCherry* construct (via *Xba*I and *Spe*I) to yield pGIPZ_CMV*mCherry*. The IRES/puromycin resistance region was extracted from the plasmid (via *Not*I and *Mlu*I) and substituted with the miR30 flanks produced by conventional PCR. The *Eco*RI restriction site encoded in the miR30 flank sequence was deleted by PCR. To do this, the 3′-miR30 region was amplified without the *Eco*RI sequence, and the original 3′-miR30 region was substituted with the ΔEcoRI amplicon (via *Bam*HI and *Mlu*I restriction sites). All plasmid constructs were maintained in *Escherichia coli* (strain Sfbl3; Invitrogen). Virus-cassette sequences were verified by automated, capillary (Sanger) sequencing.

Virus was produced in TLA-HEK293 cells using the Trans-Lenti shRNA Packaging Kit (Open Biosystems) with calcium phosphate. Virus titres were estimated by quantitative PCR^50^ using primers F (5′- TGGACAGGGGCTCGGCTGTT -3′) and R (5′- TCCGCTGGATTGAGGGCCGA -3′), following the removal of residual plasmid DNA by treatment with *DNa*seI (NEB). The functional virus titre of the calibrator (that is, EV control virus) was determined by calculating fluorescent cell colonies according to the manufacturer’s instructions (Open Biosystems). Resultant regression fit equations were used to estimate unknown titres; generally, virus titres of 1–5 × 10^6^ transducing units per ml were used.

### Cloning and validation of shRNAmir constructs

Novel, mature, artificial miRNA sequences encoded in the antisense strand of the shRNAmir stem and targeting coding regions of relevant messenger RNAs were inferred using the algorithm DSIR^51^. Each shRNAmir was designated according to the binding site in the mature sequence of the respective mRNA target. For each target gene, various shRNAmir sequences were initially selected and individually cloned into the lentiviral shRNAmir expression vector^27^,^52^ via *Xho*I and *Bam*HI sites. The sequence of each cloned insert was verified by Sanger sequencing before testing in COS7 cells.

The full-length cDNA sequences of *omega-1*, *ipse* and *kappa-5*, spanning the signal peptide-coding region and 3′-untranslated region (3′-UTR), were individually PCR-amplified from cDNA and introduced into the mammalian expression vector pcDNA3.1 myc-His (Invitrogen) via *Hin*dIII and *Xba*I. Insertions were each verified by sequencing. Protein-expressing cell lines were established by transfecting COS7 cells with linearized pcDNA3.1 plasmids using the Optifect reagent (Invitrogen) and selected using G418 (Invitrogen) over 7–10 days. Clones were analysed for transgene transcription by conventional PCR from cDNA. Next, the functionality of individual shRNAmirs was assessed by transfecting COS7 cell lines with lentiviral vector plasmids encoding shRNAmirs or the empty (control) plasmid using Lipofectamine LTX PLUS reagent (Invitrogen). Target gene transcription was assessed 48 h after transfection by qPCR^53^.

### Inference of shRNAmir target sequence specificity

Genes in the *S. mansoni* genome (http://www.genedb.org/Homepage/Smansoni) were screened for potential off-target sites for the six mature artificial miRNAs used in this study (that is, *omega-1* shRNAmir-511 and shRNAmir-557; *ipse* shRNAmir-195 and shRNAmir-384; *kappa-5* shRNAmir-616 and shRNAmir-638) based on homology to annotated 3′-UTRs; 3′-UTR sequences sharing complementarity to the mature artificial miRNA ‘seed’ region (nt 2–8) were predicted to be potential off-target sites^54^,^55^,^56^. The sequences for the mature artificial miRNAs (5′–3′) used here were as follows: UUGAUUGUAUCAUAUUGUCUG (shRNAmir-511), UGCAGAACCAUUGUAACCGAA (shRNAmir-557), UAUGGACGCUCUCUCUUCGUG (shRNAmir-195), UAGAAUCGACAGUAUGUCCUU (shRNAmir-384), UUUAGUUCGGUGUAUCUUCAU (shRNAmir-616) and UUGACCUACAGUCAACCUCGG (shRNAmir-638).

### Transduction of *S. mansoni* eggs

Following their isolation from mouse livers^48^, schistosome eggs were washed extensively in sterile PBS; individual batches of 3,000 eggs were then exposed to lentivirus particles at a multiplicity of infection of 10, or were not exposed (WT control) in 500 μl of serum-free DMEM (Gibco) and 8 μg ml^−1^ of polybrene (Sigma). Virus without shRNAmir stem (that is, EV) or viruses each containing the gene-specific shRNAmir stems *omega-1* shRNAmir-511 or shRNAmir-557; *ipse* shRNAmir-195 or shRNAmir-384; and *kappa-5* shRNAmir-616 or shRNAmir-638 were used. After an incubation for 24 h at 37 °C in 5% CO_2_, individual egg batches were washed extensively in PBS (37 °C) to remove virus particles and polybrene. Then, individual egg batches (*n*=3,000) were cultured (for 2–9 more days) in DMEM with 10% FCS and 2 mM L-glutamine and egg genomic DNA isolated with TRIzol (Invitrogen). Subsequently, the presence of viral DNA was verified by conventional PCR using specific primer sets and Southern blot analysis^57^ of genomic DNA of *S. mansoni*. The maturity and viability of eggs was monitored daily by microscopic examination. Hatching of eggs was induced 10 days after virus exposure and monitored microscopically.

### Assessing gene knockdown following transduction

The knockdown of target gene transcription in cultured *S. mansoni* eggs was assessed by qPCR. To do this, total RNA was isolated from individual batches of 3,000 eggs using TRIzol (Invitrogen) and residual DNA removed by *DNa*se I treatment; cDNA was synthesized from 250 ng of RNA by reverse transcription using random primers and MMLV reverse transcriptase (Bioline). Following the calculation of PCR efficiencies^58^, which ranged between 1.9 and 2.1, the relative transcription (R) of individual target genes was related to EV control samples (that is, mean Ct value), and transcription was normalized against those of *cox-1*, *ef1-a* and *psdm-4* after 3 days of *in vitro* culture; or *act-b*^59^ after 10 days of *in vitro* culture, according to the efficiency-corrected calculation model for multiple samples^60^. The suitability of *cox-1*, *ef1-a* and *psdm-4* was verified using the geNorm tool of the qbase^PLUS^ software (Biogazelle), employing established principles^61^.

### Mouse experimentation and pathological changes

Following the verification of gene knockdown, virus-treated and untreated egg batches were introduced into mice under sterile condition to then assess alterations in their lungs and sera. To do this, 1,000 eggs in 100 μl of sterile PBS were injected into the lateral tail vein of female BALB/c mice (7–8 weeks of age)^15^. Individual mice in treatment groups were injected with eggs previously transduced with lentiviruses containing gene-specific shRNAmir stems (*omega-1*, *ipse* or *kappa-5*). Mice in control groups were each injected with PBS (naive), untreated eggs (WT) or with eggs previously transduced with a lentivirus lacking the shRNAmir stem (that is, EV). Mice were euthanized 15 days after injection; immediately, individual lungs were perfused with 10 ml of sterile PBS (4 °C) via the heart, after which they were extracted from the thoracic cavity, and connective tissue and mediastinal lymph nodes trimmed off. Then, four investigatory components were conducted:

First, from individual right-lung lobes, single-cell suspensions were prepared^62^. Residual erythrocytes were lysed using ACK solution (Gibco) and cells resuspended in PBS containing 1% w/v of BSA. Total leukocyte counts were established by flow cytometry (FACS Calibur, BD Bioscience, USA) using Countbright absolute counting beads (Invitrogen); dead cells were excluded using 7-amino-actinomycin D (Sigma). Lung cell suspensions were incubated in FACS-staining buffer (PBS containing 1% of BSA and 5 mM of EDTA) with LEAF-purified anti-mouse CD16/32 (clone 93; BioLegend) and the following antibodies (BioLegend): allophycocyanin-conjugated anti-CD11c (clone N418, eBioscience), -CD3e (clone 145-2C11); R-phycoerythrin-conjugated anti-CD4 (clone RM4-5, Invitrogen), -F4/80 (clone BM8), -Siglec-F (clone E50-2440, BD Bioscience); fluorescein isothiocyanate-conjugated anti-B220 (clone RA3-6B2), -Ly-6G/Ly-6C (RB6-8C5). All antibodies were used at a 1:200 dilution, except anti-mouse F4/80 (1:100). Pooled cells from all mice of individual groups were also stained with antibody mixes for the respective isotypes. The LIVE/DEAD fixable Red stain kit (Invitrogen) was used to exclude dead cells. Flow cytometric acquisitions were carried out with a FACS Calibur and CellQuest software (BD) and analysed with FlowJo (Tree Star) (Cell gating; see Supplementary Fig. 2). Second, left-lung lobes were subjected to histopathological examination. Individual lobes were fixed in 10% normal-buffered formalin (pH 7.4) or embedded in Tissue-Tek OCT compound (Sakura Finetek), serially sectioned (5–8 μm), stained with haematoxylin–eosin or Masson’s Trichrome stain and then mounted in DPX (Sigma). Samples were examined for granulomata containing a centrally sectioned egg using a BX60 microscope (Olympus) equipped with a SPOT camera (Diagnostic Instruments) and Image-Pro software (MediaCybernetics). Subsequently, for individual granulomata, the granuloma area and the egg area were measured by computerized morphometry and the ratio between the two areas calculated. Third, total IgE levels in sera from individual mice were measured using the Ready-Set-Go! mouse IgE ELISA kit (eBioscience), according to the manufacturer’s instructions, using a Synergy H1 Hybrid Reader (BioTek). Fourth, lung cell suspensions were restimulated with SEA (20 μg ml^−1^) for 72 h, and culture supernatants assayed using the Mouse Th1/Th2/Th17/Th22 13-plex Kit FlowCytomix (eBioscience).

### Statistical analyses

Treatment groups were analysed for significant differences using the Kruskal–Wallis one-way analysis of variance (*P*<0.05) and Dunn *post*-*hoc* tests^63^ in relation to the EV control group (*n*=5–7) using Prism software (Graph-Pad Software Inc.).

## Author contributions

J.H. conceived and conducted the study, under the supervision of R.B.G. and B.H.K., with support from N.D.Y., A.L.E., C.N.P., C.S. and J.-P.Y.S.; J.H. and R.B.G. drafted the text and J.-P.Y.S., N.D.Y. and R.B.G. edited the manuscript, with inputs from other authors.

## Additional information

**How to cite this article:** Hagen, J. *et al*. *Omega-1* knockdown in *Schistosoma mansoni* eggs by lentivirus transduction reduces granuloma size *in vivo*. *Nat. Commun.* 5:5375 doi: 10.1038/ncomms6375 (2014).

## Supplementary Material

Supplementary Figures Supplementary Figures 1-2.

Supplementary Movie Vitality of miracidia within eggs.
