# Supplementary material for: Omega-1 knockdown in Schistosoma mansoni eggs by lentivirus transduction reduces granuloma size in vivo
Source: Nat Commun. 2014 Nov 17;5:5375. doi: 10.1038/ncomms6375 (PMC4243216; doi:10.1038/ncomms6375)
Supplement: Supplementary Figures — 1-2. [file ncomms6375-s1.pdf]

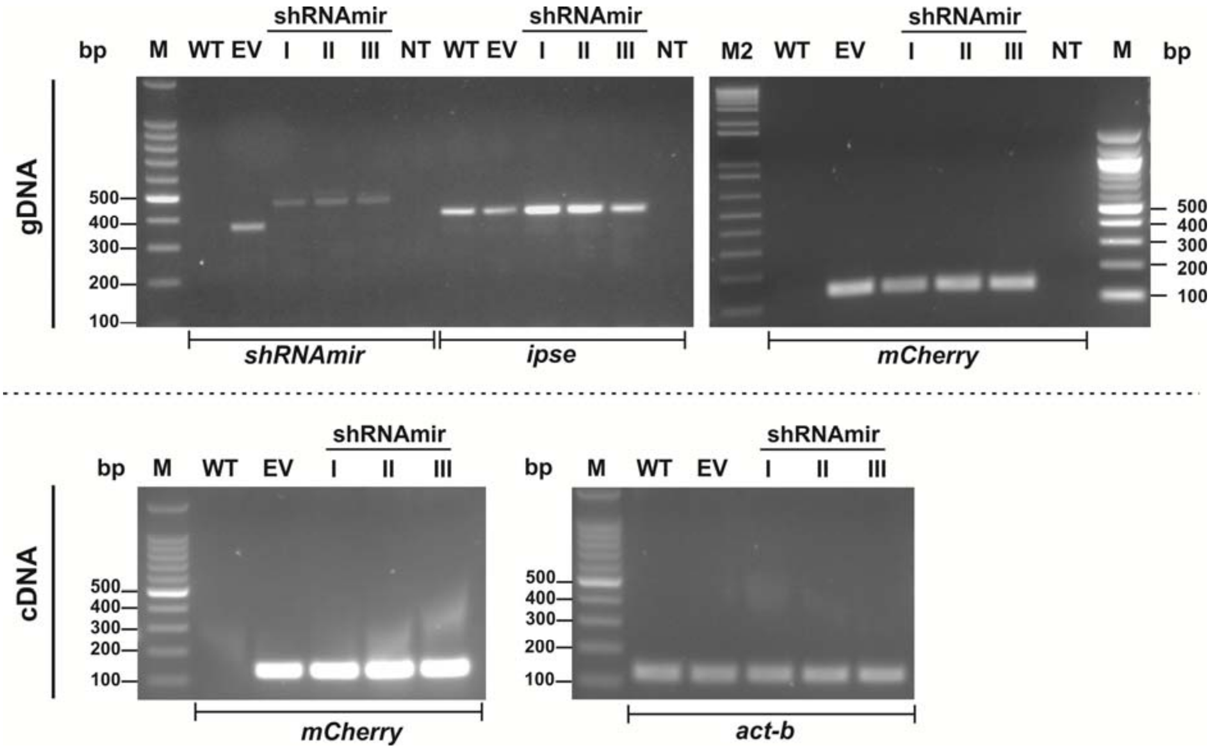

Supplementary Figure 1 | Amplification of virus regions in genomic and complementary DNA from transduced eggs of *Schistosoma mansoni*. Genomic DNA (gDNA) and RNA were isolated from transduced eggs 10 days after transduction. Lentiviral DNA was detected in gDNA by direct PCR-amplification of transgene regions, using the gene *ipse* as a positive control; cDNA was produced by reverse transcription from total RNA from eggs. Transcription of the transgene was demonstrated by direct PCR of mCherry transgene regions from cDNA, employing the actin-beta (*act-b*) coding region as a positive control. Lanes: M (molecular weight marker in bp); M2 (molecular weight marker in kb); WT (wildtype = WT); EV (empty vector control); I (shRNAmir-511+shRNAmir-557 to *omega-1*); II (shRNAmir-195+shRNAmir-384 to *ipse*); III (shRNAmir-616+shRNAmir-638 to *kappa-5*). Panels: shRNAmir = microRNA-adapted short hairpin RNA; NT (no template control).

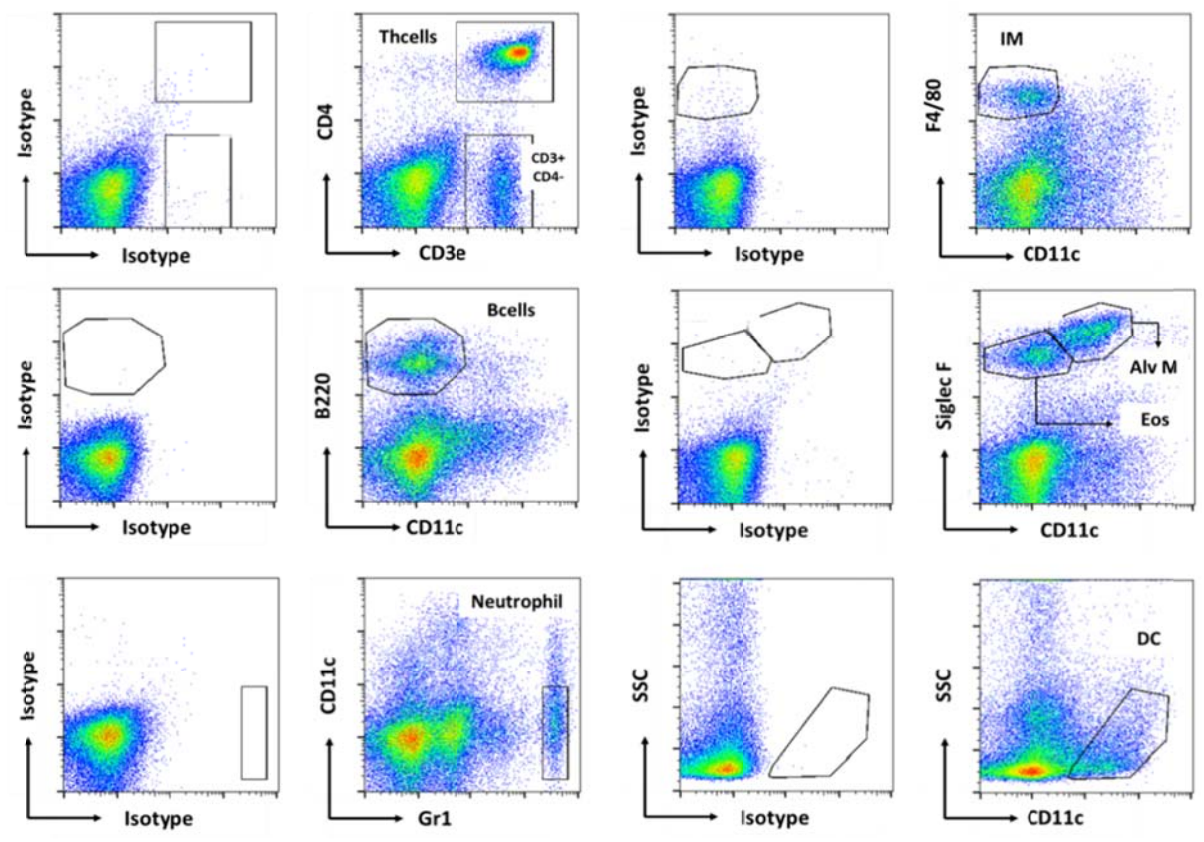

Supplementary Figure 2 | Examples of cell gating employed for the fluorescence-activated cell sorting (FACS) analyses of lung-associated leukocytes from mice. Lung cell suspensions from BALB/c mice were analysed for lung-associated leukocytes 15 days after injection (via the tail vein) with *Schistosoma mansoni* eggs. Presented are plots representing lung cell suspensions from different *S. mansoni* egg-exposed mice. Leukocytes in the live gate (LIVE/DEAD fixable red negative) were analysed by flow cytometry for expression of the surface markers CD3e+CD4+ (T helper cells), B220+CD11c- (B cells), Gr1highCD11c- (neutrophils), SiglecF+CD11c- (eosinophils, Eos), SiglecF+CD11c+ (alveolar macrophages, Alv M), F4/80+CD11c- (interstitial macrophages, IM) and C11c+SSClow (dendritic cells, DC).
